# Distribution model and prediction of the tree fern *Alsophila costularis* Baker (Cyatheaceae) in China

**DOI:** 10.1002/ece3.11594

**Published:** 2024-06-21

**Authors:** Zhen Wang, Ning Li, Ruixiang Xu, Zhanming Ying, Xiaoxian Ruan, Ting Wang, Wenbo Liao, Yingjuan Su

**Affiliations:** ^1^ School of Life Sciences Sun Yat‐sen University Guangzhou China; ^2^ College of Chemistry, Xiangtan University Xiangtan China; ^3^ Research Institute of Sun Yat‐sen University in Shenzhen Shenzhen China; ^4^ College of Life Sciences, South China Agricultural University Guangzhou China

**Keywords:** *Alsophila costularis* Baker, climate change, environmental variables, MaxEnt model, potentially suitable habitat, species distribution models

## Abstract

Climatic change is a challenge for plant conservation due to plants' limited dispersal abilities. The survival and sustainable development of plants directly depend on the availability of suitable habitats. In this study, we employed an optimized MaxEnt model to evaluate the relative contribution of each environmental variable and predict the suitable habitat for *Alsophila costularis* under past, current, and future periods, which is an endangered relict tree fern known as a living fossil. For the Last Glacial Maximum (LGM) and Mid‐Holocene scenarios, we adopted two atmosphere–ocean general circulation models: CCSM4 and MIROC‐ESM. The BCC‐CSM2‐MR model was used for future projections. The results revealed that temperature annual range (Bio7) contributed most to the model construction with an optimal range of 13.74–22.44°C. Species distribution modeling showed that current suitable areas were mainly located in most areas of Yunnan, most areas of Hainan, most areas of Taiwan, southeastern Tibet, southwestern Guizhou, western Guangxi, southern Sichuan, and southern Guangdong, with an area of 35.90 × 10^4^ km^2^. The suitable habitat area expanded northward in Yunnan from the Last Interglacial to the LGM under the CCSM4 model, while a significant contraction toward southwestern Yunnan was found under the MIROC‐ESM model. Furthermore, the potential distributions during the Mid‐Holocene were more widespread in Yunnan compared to those under current period. It is predicted that in the future, the range will significantly expand to northern Yunnan and western Guizhou. Almost all centroids of suitable habitats were distributed in southeastern Yunnan under different periods. The stable areas were located in southwestern Yunnan in all scenarios. The simulation results could provide a theoretical basis for the formulation of reasonable conservation and management measures to mitigate the effects of future climate change for *A. costularis*.

## Graphical abstract

We employed an optimized MaxEnt model to evaluate the relative contribution of each bioclimatic variable and predict the suitable habitat for *Alsophila costularis* Baker under past, current, and future climate scenarios, which is an endangered relict tree fern known as a living fossil. The results revealed that temperature annual range (Bio7) contributed most to the model construction with an optimal range of 13.74–22.44°C. Species distribution modeling showed that current suitable areas were mainly located in most areas of Yunnan, most areas of Hainan, most areas of Taiwan, western Guangxi, and southern Guangdong in China, with an area of 35.90 × 10^4^  km^2^.

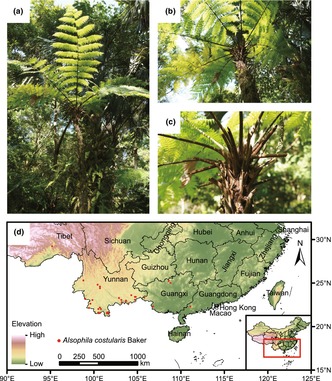

## 1 INTRODUCTION

Climate can influence the reproduction and growth of organisms, as well as shape the distribution ranges of plants (Akyol et al., 2020). The global climate was characterized as colder and drier during the Last Glacial Maximum (LGM, about 21 kilo years ago (kya)), after which it began to warm (Nogués‐Bravo et al., 2010). Under the influence of glaciation, the temperature in southern China decreased by about 4–6°C, precipitation reduced by 400–600 mm compared to the current period, and the mountain forest boundaries were lowered (Zheng et al., 1998). Drastic climate cooling forced species to shrink into refugia during the Last Glacial Maximum (Bai et al., 2018). Subsequently, with the warming of the climate, plants began to colonize areas outside of the refugia (Normand et al., 2011).

Under continued global warming, the mean surface temperature will increase by 1.1–6.4°C at the end of the century (Gao et al., 2023). Global warming can lead to the fragmentation or even complete loss of plant habitats. However, some plants may migrate to new suitable habitats in order to adapt to climate change by shifting their climatic niche (Xie et al., 2022). As influenced by directional selection and rapid migration, climate change could reduce the genetic diversity of populations, thereby impacting ecosystem functioning (Bellard et al., 2012). Hence, modeling how the potential distribution of species responds to climate change over time can provide a scientific basis for biodiversity conservation.

Species distribution models assess the niche requirements of species by correlating occurrence records with environmental variables and ultimately produce suitability maps. These maps are a widely used tool for delineating species conservation regions (Esselman & Allan, 2011; Fourcade et al., 2018). However, species distribution models assume that the niche of species and biotic interactions remain constant over time, which may be unrealistic, especially for long time scales (Roberts & Hamann, 2015). Therefore, we should be judicious in our results when projecting the potential distribution of species for historical or future periods.

As one of the most popular software for constructing species distribution models, MaxEnt is used in numerous studies (Merow et al., 2013). MaxEnt is a maximum entropy‐based method that estimates the habitat suitability of the species based on presence‐only distribution records and environmental constraints (Phillips et al., 2006). This approach is widely employed to generate the distribution model and provides a useful tool for predicting the geographic distribution of species with few occurrence sites, making it an ideal software for studying potentially suitable habitats of endangered species (Elith et al., 2006; Gao et al., 2022; Kong et al., 2021).

**FIGURE 1:**
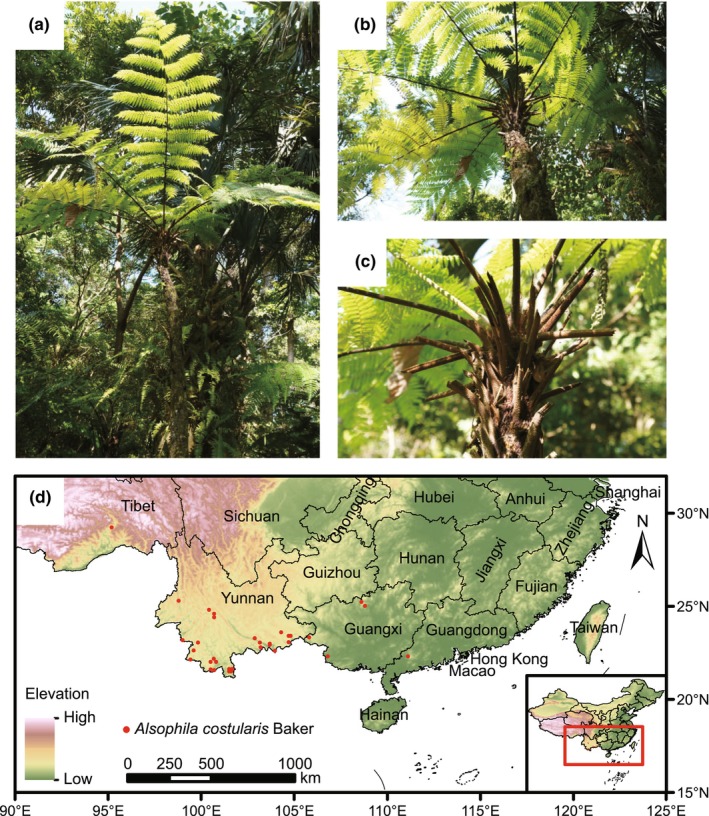
Photographs (a–c) and occurrence records (d) of *Alsophila costularis* used in the MaxEnt model. All photographs were taken by Guohua Zhao.

*Alsophila costularis* Baker (Cyatheaceae) is an endangered relict tree fern known as a living fossil (Ying et al., 2009). Its trunk is erect with a height that can exceed 5 m (Figure 1). The stipe has scattered glossy dark brown scales with fragile edges. Its spore germination and gametophyte development require strict environmental conditions (Cheng et al., 1990; Song et al., 2008). The species prefers to grow in warm and moist environments, and canyon forest habitats provide suitable hydrothermal conditions and favorable topographic shade (Yuan et al., 2023; Zhang & Nishida, 2013). *Alsophila costularis* has important ornamental and medicinal values, and is often deforested excessively (Ying et al., 2009). As a result, *A. costularis* is listed as a national second‐class protected plant in China and in the Convention on International Trade in Endangered Species (CITES) (Oldfield, 1995; Zhang et al., 2011).

*Alsophila costularis* inhabits ravine forests at 700–2100 m a.s.l. (Zhang & Nishida, 2013), which is sensitive to soil contamination. The species can germinate normally when the concentration of Pb is less than 10^−5^ mol/L (Wang et al., 2015). In China, it is distributed in Guangxi, Tibet, and Yunnan, and elsewhere in Bangladesh, Bhutan, India, Myanmar, and Vietnam (Figure 1) (Zhang & Nishida, 2013). Like all vascular plants, *A. costularis* has two independent generations, a haploid gametophyte and a diploid sporophyte. It is homosporous and produces abundant small, wind‐dispersed spores. Compared to other ferns of Cyatheaceae, *A. costularis* has been little studied, especially as regards its ecology. Previous studies have focused on tissue culture (Cheng & Liu, 1992), gametophyte development (Wang et al., 2007; Xiao et al., 2019), DNA extraction (Ying et al., 2009), chloroplast genome (Wang et al., 2019), and the efficient green globular bodies system (Pu et al., 2023).

*Alsophila costularis* is primarily native to Yunnan Province in China, where it persists in small and isolated populations. These populations are considered a refugium due to the lack of impact of Quaternary glaciers (López‐Pujol et al., 2011). Climate is one of the major factors affecting plant distribution (Pauls et al., 2013). However, it is unknown which climatic factors have played important roles in forming the distribution of *A. costularis* in the past and present and how will the distribution change in the future. Ecological niche modeling can provide new insight into these questions by predicting the occurrence of species and distinguishing the contributing climatic factors (Lozier et al., 2009). Combined with occurrence records of the species and environmental variables, reliable distribution predictions can be acquired.

In this study, we used the MaxEnt model to investigate the potential distribution of *A. costularis* in China. The study aimed at (i) revealing which climatic or environmental factors exert the greatest impact on the potential distribution, and (ii) predicting the past, current, and future ranges and centroids of suitable habitat of *A. costularis*, and assessing the impact of environmental change in the LGM on the refugium of the species in Yunnan. The research goal is to provide a theoretical basis for the conservation and management of *A. costularis*.

## 2 MATERIALS AND METHODS

### 2.1 Collecting species occurrence records

We collected 37 species occurrence records from field surveys, the published literature, the Global Biodiversity Information Facility (GBIF, https://www.gbif.org/), the National Specimen Information Infrastructure (NSII, http://www.nsii.org.cn/), and the Chinese Virtual Herbarium (CVH, https://www.cvh.ac.cn/). The spThin v0.2.0 package in R v4.3.2 (R Core Team, 2023) was used to remove records <5 km apart (Aiello‐Lammens et al., 2015), a distance equal to the resolution of the environmental layer (2.5 arc‐min), to avoid the effects of spatial autocorrelation (Gómez‐Rodríguez et al., 2015). As a result, 33 unique occurrence records were saved in .csv format and used in the modeling for *A. costularis* (Figure 1d).

### 2.2 Extracting environmental variables

The potential distribution of species is influenced by a variety of environmental factors, such as climatic variables, soil factors, and ultraviolet‐B radiation. We selected 38 environmental variables in total to predict the suitable habitat of *A. costularis*, including 19 bioclimatic variables, 13 soil variables, and six ultraviolet‐B (UV‐B) variables. The bioclimatic variables were downloaded from the WorldClim v1.4 database (https://www.worldclim.org/) (Hijmans et al., 2005) for the past periods with 2.5 arc‐min spatial resolution, including the Last Interglacial (LIG, about 140–120 kya), LGM, and Mid‐Holocene (MH, about 6 kya). For LGM and MH layers, we adopted two atmosphere–ocean general circulation models based on the Coupled Model Intercomparison Project Phase 5 (CMIP5) protocol: Community Climate System Model version 4 (CCSM4) (Gent et al., 2011) and Model for Interdisciplinary Research on Climate‐Earth System Model (MIROC‐ESM) (https://www.worldclim.org/) (Watanabe et al., 2011). In addition, we obtained the current (1970–2000) and future (2050s, 2070s, and 2090s) bioclimatic variables from the WorldClim v2.1 database (https://www.worldclim.org/) (Fick & Hijmans, 2017) at a spatial resolution of 2.5 arc‐min. The 2050s, 2070s, and 2090s climate data represent average values from 2041 to 2060, 2061 to 2080, and 2081 to 2100, respectively. The Beijing Climate Center‐Climate System Model‐Medium Resolution (BCC‐CSM2‐MR) climate model (Wu et al., 2019) was used to simulate future geographical distribution of *A. costularis* under four different Shared Socioeconomic Pathways (SSPs) (O'Neill et al., 2014) of the Coupled Model Intercomparison Project Phase 6 (CMIP6), including SSP1‐2.6, SSP2‐4.5, SSP3‐7.0, and SSP5‐8.5. The SSP1‐2.6 and SSP5‐8.5 separately demonstrate low and high gas emissions, respectively, whereas SSP2‐4.5 and SSP3‐7.0 represent medium scenarios for gas emission (Riahi et al., 2017). To ensure consistency with current and future temperature data, we divided the values of all original paleoclimate temperature layers (except Bio3) by ten using ArcGIS v10.2 (ESRI, Redlands, CA, USA). Moreover, 13 soil factors were accessed from the National Tibetan Plateau Data Center (https://data.tpdc.ac.cn) with a resolution of 1000 m at a depth of 15–30 cm (Liu et al., 2022). We downloaded six UV‐B variables from the global UV‐B radiation dataset (https://www.ufz.de/gluv/) at a 15 arc‐min spatial resolution (Beckmann et al., 2014). All environmental layers were clipped to the approximate distribution range (15–32°N, 90–125°E) of *A. costularis* with the same projection system and cell size and then transformed into ASCII format by using ArcGIS v10.2 (ESRI, Redlands, CA, USA). Soil factors and UV‐B variables were assumed to keep constant under past and future periods due to the absence of data (Mengistu et al., 2023).

Currently, a variety of methods can be used to detect the multicollinearity of variables, such as the Pearson's correlation coefficient. However, a Pearson's correlation coefficient of 0.28 can lead to the model overfitting (Graham, 2003). Therefore, the variance inflation factor (VIF) was calculated using usdm v2.1.7 package (Naimi et al., 2014) in R to examine the multicollinearity among 38 environmental variables. Meanwhile, we calculated the regularization training gain using the jackknife test in MaxEnt v3.3.3 k (Phillips et al., 2006). Finally, ten variables with VIF < 5 and the regularization training gain greater than 0.1 were employed for ecological niche modeling (ENM), including Bio7 (Temperature annual range), Bio8 (Mean temperature of wettest quarter), Bio13 (Precipitation of wettest month), Bio18 (Precipitation of warmest quarter), Bio19 (Precipitation of coldest quarter), btcly (Clay content), btslt (Silt content), socd (Soil organic carbon density), texcls (Texture classifications), and UVB3 (Mean UV‐B of highest month) (Table 1). These selected predictive variables reflect the annual trends and extremes of temperature, precipitation, or UV‐B radiation.

**TABLE 1:** Summary of variance inflation factor (VIF), percentage contribution, and permutation importance for ten environmental variables.

Code	Environmental variable	Variance inflation factor	Percentage contribution (%)	Permutation importance (%)
Bio7	Temperature annual range	2.24	44.9	24.4
Bio18	Precipitation of warmest quarter	4.56	15.0	0.1
texcls	Texture classifications	1.94	11.8	8.8
UVB3	Mean UV‐B of highest month	2.51	11.0	15.7
Bio13	Precipitation of wettest month	4.19	9.2	37.0
Bio19	Precipitation of coldest quarter	2.25	4.5	8.8
btslt	Silt content	1.69	1.6	2.9
Bio8	Mean temperature of wettest quarter	3.84	1.3	0.1
btcly	Clay content	3.24	0.7	2.2
socd	Soil organic carbon density	2.80	0.0	0.0

### 2.3 Constructing, optimizing, and evaluating species distribution models

To assess the environmental suitability of *A. costularis*, we used ecological niche modeling to predict its potential geographic distribution through MaxEnt v3.3.3 k in seven different periods. The species distribution models (SDMs) were constructed by importing unique occurrence records and selected environmental variables into MaxEnt with ten cross‐validation replicates, the maximum number of background points of 10,000, and the maximum iterations of 500. The occurrence records were split into ten subsets, and nine subsets were used as the training dataset, while the remaining one subset was employed to evaluate the model predictions for each replication. Moreover, the predicted suitability was represented by logistic value. The predictive performance of the MaxEnt model is significantly determined by the regularization multiplier (RM) and feature classes (FC) (Phillips & Dudík, 2008). Model accuracy is affected by the fact that the type and complexity of the environmental dependencies. These dependencies are characterized by simple functions generated from the environmental variables, which are referred to as “features” (Phillips & Dudík, 2008). Therefore, to reduce the overfitting and complexity of the model, the regularization multiplier and feature classes were regulated using the ENMeval v2.0.4 package (Kass et al., 2021) in R. We set the regularization multiplier values to 0.5–5.0 with each interval of 0.5. Six feature combinations were adopted: L, LQ, H, LQH, LQHP, and LQHPT, where L = linear, Q = quadratic, H = hinge, P = product, and T = threshold. As a result, 60 parameter combinations were employed in the ENMeval analysis. The fit and complexity of the model were evaluated based on Akaike information criterion correction (AICc) and delta Akaike information criterion correction (delta.AICc) (Burnham & Anderson, 2004). The parameter combination with the lowest AICc value was selected for further model prediction. Based on the current climatic conditions, the species distribution model was constructed using the optimized parameters. Then, the model obtained was projected onto environmental layers for LIG, LGM, MH, 2050s, 2070s, and 2090s. We calculated the suitability curves of each variable and their contributions to species distribution using the jackknife test, percent contribution, and permutation importance.

The model performance was assessed with the Jaccard's similarity index and Sørensen's similarity index (Leroy et al., 2018). The Jaccard's similarity index and Sørensen's similarity index were estimated by equations Jaccard = TP/(FN + TP + FP) and Sørensen = 2TP/(FN + 2TP + FP), where TP is true positives, FN is false negatives, and FP is false positives. The range of similarity indices is 0–1. The similarity indices of 0 suggest a complete mismatch between actual observations and predictions of the species, while a value of 1 indicates that the predictions are perfectly matched to observations (Leroy et al., 2018). The similarity indices not <0.7 indicate an acceptable threshold (Sundar et al., 2021). We selected the maximum training sensitivity plus specificity threshold (MTSS) (Liu et al., 2005) as the threshold value of the binary presence/absence model. Liu et al. (2005) compared 12 thresholds that convert species distributional suitability into presence/absence map and showed that MTSS was recommended. We use ArcGIS software to convert output layers of MaxEnt in ASCII format into raster layers and to divide the potential distribution ranges into two categories according to occurrence suitability, including unsuitable habitat (<MTSS) and suitable habitat (>MTSS). Furthermore, the area of suitable habitat was estimated based on the number and size of grid cells.

### 2.4 Shifts in distribution area and distribution centroid of suitable habitat

We reclassified the continuous occurrence probabilities into binary presence/absence maps by applying MTSS within ArcGIS. The SDMtoolbox v2.10 toolkit (Brown et al., 2017) was employed to analyze the changes in the area of suitable habitat between current and other periods with Distribution Changes Between Binary SDMs. In addition, centroid shifts of suitable habitat were evaluated using SDMtoolbox v2.10 toolkit with Centroid Changes under different climatic conditions to trace the migration direction of *A. costularis*.

### 2.5 Identifying stable areas

Stable areas are regions that are continuously suitable for species distribution over different periods of time. To identify the stable areas, we used the raster calculator in ArcGIS to create a composite map by overlaying the suitable habitat maps for *A. costularis* across all scenarios. In addition, all protected areas in China were downloaded from the World Database on Protected Areas (WDPA) (https://www.protectedplanet.net/en) and overlapped on the stable areas to determine the effectiveness of protected areas. If stable areas overlap with protected areas, this is an indication that conservation efforts are highly effective, and conversely, the establishment of new protected areas needs to be considered.

## 3 RESULTS

### 3.1 Optimal model and MaxEnt model evaluation

The ENMeval test suggested that the optimal MaxEnt model was obtained for the parameter combination of the feature classes H and the regularization multiplier of 2.0 with the lowest AICc value and delta.AICc value of zero (Figure S1), which could decrease the model complexity. Therefore, the optimized parameter combination (RM = 2.0 and FC = H) was used to construct the species distribution models. At this point, the Jaccard's similarity index and Sørensen's similarity index of ten cross‐validation replicates were 0.788 ± 0.033 and 0.881 ± 0.020, respectively, which indicated that predictions were ideally matched to observations of species and the current distribution of *A. costularis* based on the selected variables was accurate.

### 3.2 Contribution assessment of environmental variables

Based on the jackknife test, several environmental factors emerged as important variables influencing the distribution of *A. costularis*, when only one variable was used (Figure S2). Temperature annual range (Bio7) contributed most to the model with the largest regularized training gain, test gain, and AUC value, followed by precipitation of warmest quarter (Bio18), mean UV‐B of highest month (UVB3), precipitation of wettest month (Bio13), and precipitation of coldest quarter (Bio19). In brief, the jackknife test suggested that the distribution range of *A. costularis* was mainly modeled by precipitation variables. Based on the percentage contribution and the permutation importance, temperature annual range (Bio7), precipitation of warmest quarter (Bio18), texture classifications (texcls), mean UV‐B of highest month (UVB3), and precipitation of wettest month (Bio13) cumulatively contributed 91.9% to the construction of species distribution models, with percentage contribution rates of 44.9%, 15.0%, 11.8%, 11.0%, and 9.2%, respectively, and permutation importance values of 24.4%, 0.1%, 8.8%, 15.7%, and 37.0%, respectively (Table 1). In summary, temperature annual range (Bio7) and precipitation of wettest month (Bio13) were the most critical climatic variables for the suitable habitat of *A. costularis*.

The MaxEnt‐based response curves showed the optimal range of each environmental variable in predicted distribution suitability are visualized in Figure S3. The suitable climatic conditions for *A. costularis* included temperature annual range of 13.74–22.44°C, precipitation of warmest quarter greater than 668.33 mm, mean UV‐B of highest month of 5653.06–7338.84 J/m^2^/day, precipitation of wettest month greater than 277.71 mm, and precipitation of coldest quarter of 45.84–92.96 mm (suitability of presence >0.5).

### 3.3 Distribution prediction of *A. costularis* in China

**FIGURE 2:**
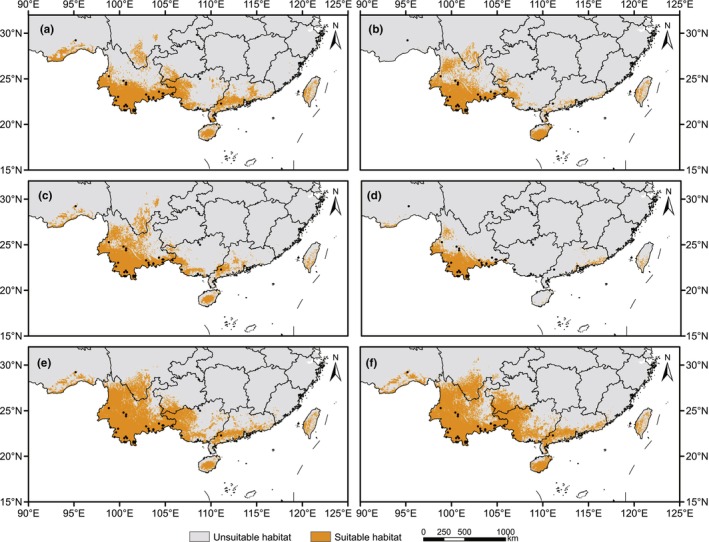
Predicted potential distribution areas of *Alsophila costularis* during current and historical periods based on ecological niche models. (a) Predicted current distribution. (b) Predicted distribution during the Last Interglacial (LIG). (c) Predicted distribution during the Last Glacial Maximum (LGM) based on Community Climate System Model version 4 (CCSM4). (d) Predicted distribution during the LGM based on Model for Interdisciplinary Research on Climate‐Earth System Model (MIROC‐ESM). (e) Predicted distribution in the Mid‐Holocene (MH) based on CCSM4. (f) Predicted distribution in the Mid‐Holocene based on MIROC‐ESM. The occurrence records used for modeling are represented by black dots.

**FIGURE 3:**
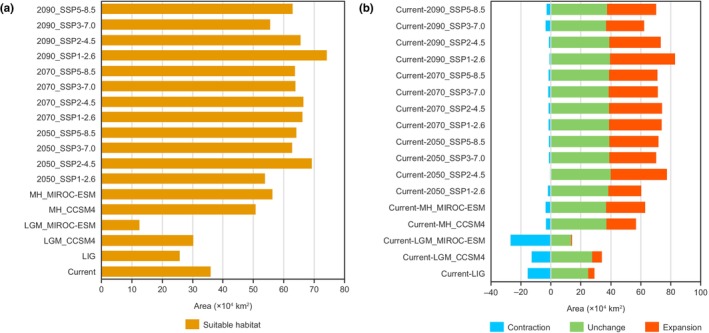
Areas (a) and area changes (b) of predicted suitable habitat in different climatic periods based on MaxEnt model.

For *A. costularis*, current suitable areas were distributed in large parts of Yunnan, Hainan, Taiwan, southeastern Tibet, southwestern Guizhou, western Guangxi, southern Sichuan, and southern Guangdong in China (Figure 2a). The total area of suitable habitat was evaluated as 35.90 × 10^4^ km^2^ with a suitability of prediction >0.211 (MTSS) in China (Figure 3a and Table 2).

**TABLE 2:** The suitable habitat areas of *Alsophila costularis* under different climatic periods based on MaxEnt prediction.

Period	Climate scenario	Predicted area (×10^4^ km^2^)
Current		35.90
LIG		25.69
LGM	CCSM4	30.22
	MIROC‐ESM	12.48
MH	CCSM4	50.77
	MIROC‐ESM	56.18
2050s	SSP1‐2.6	53.76
	SSP2‐4.5	69.23
	SSP3‐7.0	62.69
	SSP5‐8.5	64.16
2070s	SSP1‐2.6	66.02
	SSP2‐4.5	66.35
	SSP3‐7.0	63.76
	SSP5‐8.5	63.58
2090s	SSP1‐2.6	74.05
	SSP2‐4.5	65.52
	SSP3‐7.0	55.45
	SSP5‐8.5	62.85

For the historical period, the suitable areas were largely found in southwestern Yunnan, Hainan, and Taiwan (Figure 2b–f). An expansion occurred during the transition from the LIG to the LGM (Figure 2c) under the CCSM4 model, while a significant contraction was observed under MIROC‐ESM model (Figure 2d), with suitable areas increased by 4.53 × 10^4^ km^2^ for the CCSM4 model and decreased by 13.21 × 10^4^ km^2^ for the MIROC‐ESM model. In the LGM, the suitable areas contracted greatly toward southwestern Yunnan under MIROC‐ESM model, and almost all suitable habitats were lost in the remaining provinces (Figure 2d). In comparison to the LGM and current periods, the potential distribution in the Mid‐Holocene was more widespread in Yunnan (Figure 2e,f). Compared to the MIROC‐ESM model (12.48 × 10^4^ km^2^), the CCSM4 model (30.22 × 10^4^ km^2^) forecasted a wider distribution, which had a more extensive distribution in Yunnan, Hainan, Guangxi, and Guangdong (Table 2). From the LGM to the Mid‐Holocene, the historical potential ranges of the model increased significantly by 20.55 × 10^4^ km^2^ and 43.70 × 10^4^ km^2^ under CCSM4 and MIROC‐ESM models, respectively, which was located primarily in northern Yunnan, Hainan, and western Guangxi (Figure 2e,f). During the Mid‐Holocene, the suitable habitats covered Yunnan, Hainan, Taiwan, and western Guangxi with areas of 50.77 × 10^4^ km^2^ and 56.18 × 10^4^ km^2^ under CCSM4 and MIROC‐ESM models, respectively (Table 2).

**FIGURE 4:**
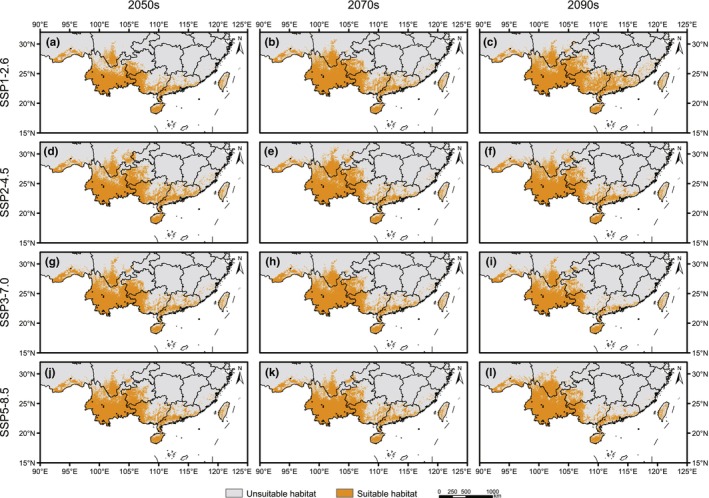
Predicted potential distribution areas of *Alsophila costularis* in the future periods based on ecological niche models. Predicted distribution under SSP1‐2.6 scenario in the 2050s (a), 2070s (b), and 2090s (c), under SSP2‐4.5 scenario in the 2050s (d), 2070s (e), and 2090s (f), under SSP3‐7.0 scenario in the 2050s (g), 2070s (h) and 2090s (i), and under SSP5‐8.5 scenario in the 2050s (j), 2070s (k), and 2090s (l). The occurrence records used for modeling are represented by black dots.

For the future environmental conditions, the model predicted that major changes would take place for *A. costularis*, including a significant increase in northern Yunnan and western Guizhou (Figure 4). In general, the suitable habitats showed a northward expansion trend under the future scenarios. Compared with the current species distribution model, the total areas of suitable habitat would be increased under all future climate change scenarios for *A. costularis* (Figure 3a and Table 2). The suitable growth areas of 12 species distribution models ranged from 53.76 × 10^4^ km^2^ to 74.05 × 10^4^ km^2^ under different SSPs in the future, all of which were increased significantly by more than 49.75%. Under the SSP1‐2.6 scenario, the suitable distributions would expand gradually from 2050s to 2090s, whereas this trend was not observed under the other three scenarios.

### 3.4 Shifts in distribution area and distribution centroid of suitable habitat

**FIGURE 5:**
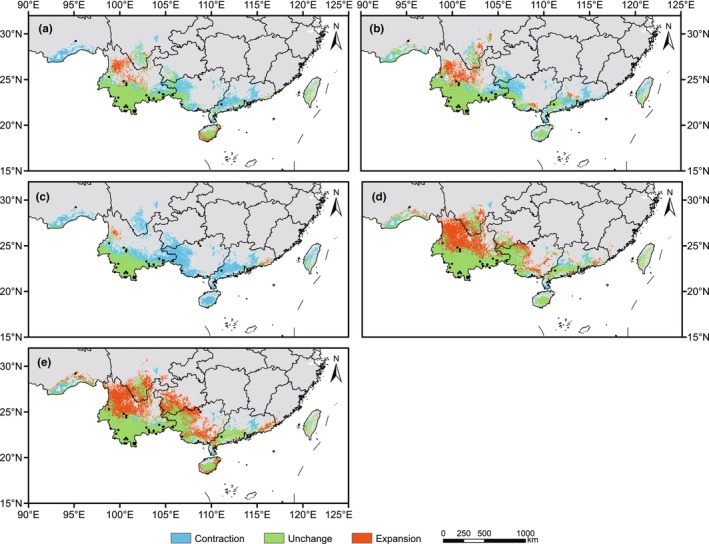
Spatial change map of potential distribution regions between current and historical periods. (a) Last Interglacial, (b) Last Glacial Maximum (Community Climate System Model version 4), (c) Last Glacial Maximum (Model for Interdisciplinary Research on Climate‐Earth System Model), (d) Mid‐Holocene (Community Climate System Model version 4), and (e) Mid‐Holocene (Model for Interdisciplinary Research on Climate‐Earth System Model).

**FIGURE 6:**
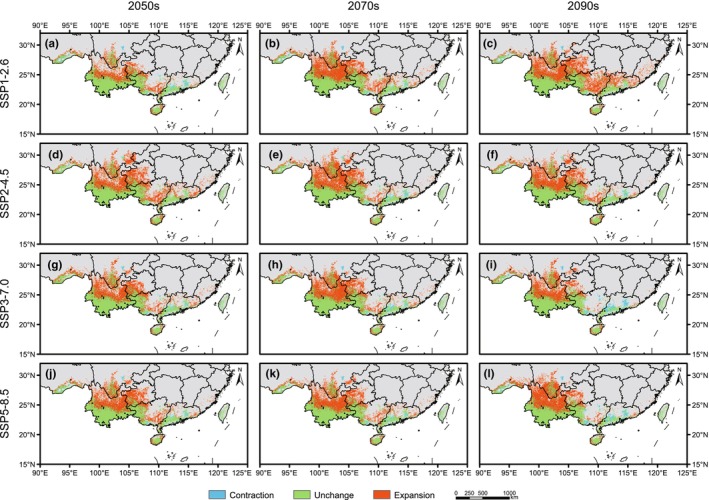
Spatial change map of potential distribution regions between current and future periods. Spatial change under SSP1‐2.6 scenario in the 2050s (a), 2070s (b), and 2090s (c), under SSP2‐4.5 scenario in the 2050s (d), 2070s (e), and 2090s (f), under SSP3‐7.0 scenario in the 2050s (g), 2070s (h), and 2090s (i), and under SSP5‐8.5 scenario in the 2050s (j), 2070s (k), and 2090s (l).

During the LIG, the suitable habitat of *A. costularis* shrank significantly, with a contraction area of 15.40 × 10^4^ km^2^ (Figure 3b and Table 3). Distribution ranges were entirely lost in southeastern Tibet, and most of the suitable habitats in western Guangxi and southern Guangdong were lost (Figure 5a). The ranges of species distribution were expanded mainly in northern Yunnan under the CCSM4 climate scenario in the LGM (Figure 5b). In contrast, the margins of suitable habitats in Yunnan, western Guangxi, southern Guangdong, Hainan, and southeastern Tibet contracted under MIROC‐ESM, and expansion was barely detected (Figure 5c). The potential suitable area expanded to the northern boundary of Yunnan and margins of suitable habitats in western Guangxi under two atmosphere–ocean general circulation models for the Mid‐Holocene period (Figure 5d,e). For future models, the suitable areas would expand significantly, while little contraction would occur (Figure 6). The expansion of habitats would be founded in northern Yunnan, southwestern Guizhou, and southern Guangxi under different SSPs. The largest expansion of suitable area occurred under the SSP1‐2.6 scenario in the 2090s period, with an area of 43.38 × 10^4^ km^2^ (Figure 3b).

**TABLE 3:** The change in suitable habitat area of *Alsophila costularis* between different climatic periods.

	Climate scenario	Expansion (×10^4^ km^2^)	Unchange (×10^4^ km^2^)	Contraction (×10^4^ km^2^)
Current‐LIG		4.19	24.82	15.40
Current‐LGM	CCSM4	6.47	27.46	12.89
	MIROC‐ESM	0.68	13.46	26.89
Current‐MH	CCSM4	19.86	37.01	3.34
	MIROC‐ESM	26.06	36.78	3.57
Current‐2050s	SSP1‐2.6	21.92	38.44	1.91
	SSP2‐4.5	37.38	39.93	0.42
	SSP3‐7.0	31.17	39.04	1.31
	SSP5‐8.5	32.74	39.03	1.32
Current‐2070s	SSP1‐2.6	35.06	38.89	1.46
	SSP2‐4.5	35.23	38.88	1.47
	SSP3‐7.0	32.64	38.65	1.70
	SSP5‐8.5	32.27	38.84	1.51
Current‐2090s	SSP1‐2.6	43.38	39.37	0.98
	SSP2‐4.5	34.11	39.09	1.26
	SSP3‐7.0	25.20	36.91	3.44
	SSP5‐8.5	32.79	37.41	2.94

**FIGURE 7:**
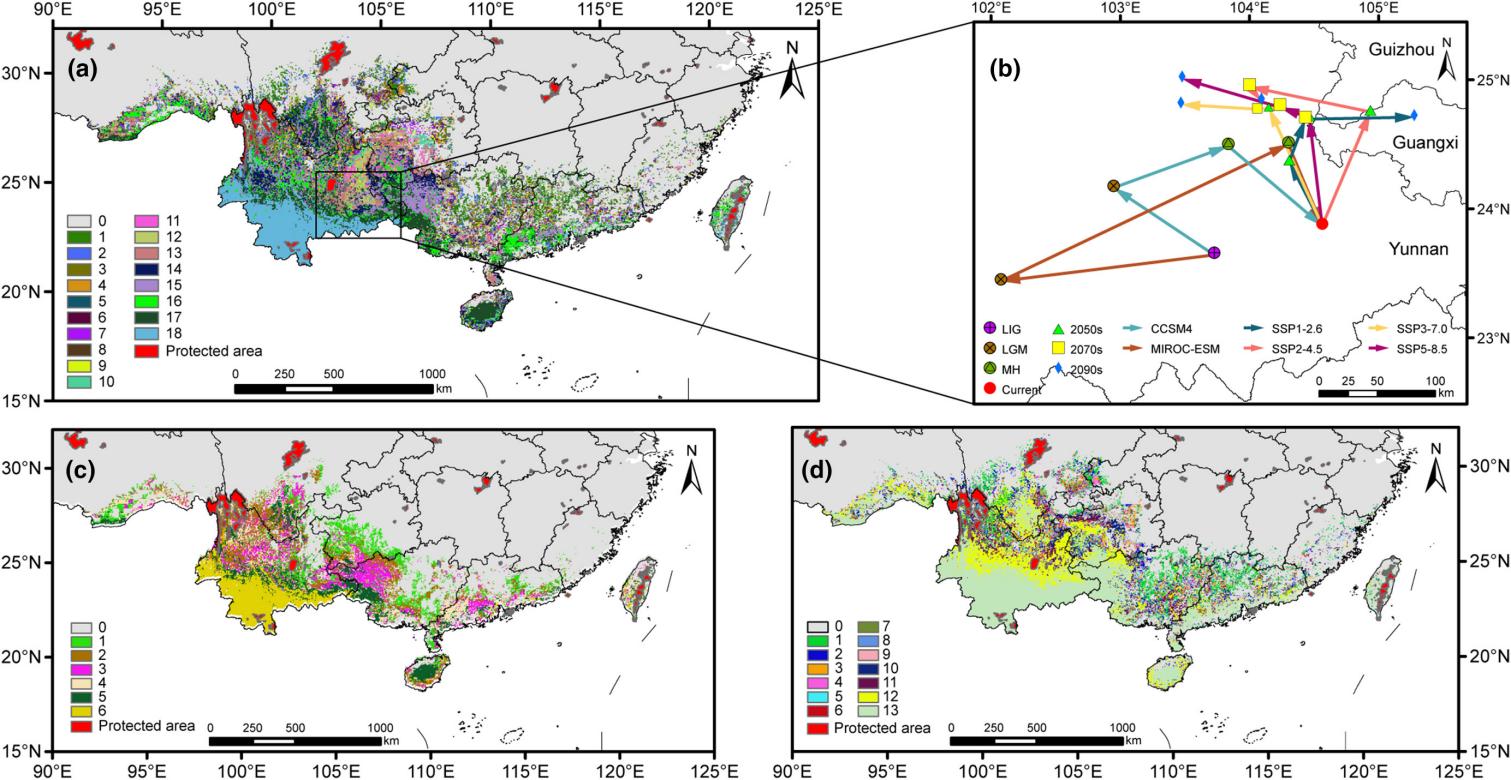
Stable areas of *Alsophila costularis* in all scenarios (a), from past to current climate scenarios (c), and from current to future climate scenarios (d), overlapping the protected areas, and migration routes of centroids of potentially suitable habitats under different climate scenarios (b). In (a, c, d), stable areas supported by various numbers of scenarios are shown in different colors.

Once the binary presence/absence map was obtained, the suitable range of the species was reduced to a single central point, known as the centroid (Brown, 2014). The centroids of suitable habitats were located in southeastern Yunnan during different periods except for the 2050s under the SSP2‐4.5 scenario and 2090s under the SSP1‐2.6 scenario (Figure 7b). During the transition from the LIG to the LGM, the centroid positions of *A. costularis* would migrate toward the northwest and southwest under the CCSM4 model and MIROC‐ESM model, respectively, and then continued to shift northeastward in the Mid‐Holocene until finally southeastward to reach the current centroid in the border of Yunnan and Guangxi (Figure 7b). However, the migration routes of centroids were more complex for the future period under four different SSPs (Figure 7b). For the SSP1‐2.6 scenario, *A. costularis* would transport their ranges northwestward from the present to the 2050s, followed by northeastward in the 2070s, and lastly eastward from the 2070s to the 2090s. For the SSP2‐4.5 scenario, the centroids of the current suitable area moved to the junction of Guangxi and Guizhou in the 2050s, then to the northwest in the 2070s, and finally to the southeast in the 2090s. Under the SSP3‐7.0 and SSP5‐8.5 scenarios, the distribution centroid shifted continuously northwestward from the current to the 2090s.

### 3.5 Identifying stable areas

The stable areas were located in southwestern Yunnan under all scenarios, past scenarios, and future scenarios, a region where most of the specimen records of *A. costularis* have been found (Figure 7a,c,d). The overlap between stable areas and protected areas enables an assessment of whether the current extent of protected areas is able to cope with the challenges posed by environmental change. The current network of protected areas failed to provide full coverage of the stable zone. The protected areas within the stable areas were found in the southernmost part of Yunnan, such as Xishuangbanna Dai autonomous prefecture.

## 4 DISCUSSION

### 4.1 Model prediction accuracy

The development of conservation and management measures relies on the reliable and accurate species distribution modeling. The MaxEnt model has been employed extensively for species distribution models, which assess the effects of environmental variation on species distribution (Feng et al., 2019). The reliability of MaxEnt results is influenced by occurrence records, variable selection, and model optimization (Schnase et al., 2021; Xie et al., 2022). We avoid the influence of spatial autocorrelation by ensuring that the distance between the occurrence records is not <5 km. Spatial filtering decreases the influence of sampling bias, which can improve the accuracy of species distribution models (Boria et al., 2014; Franklin, 2023). In addition, the bioclimatic variables with high multicollinearity were removed in the present study. We constructed MaxEnt models for tree fern *A. costularis* by optimizing the regularization multiplier and feature classes parameters under past, current, and future climatic conditions. As a result, RM = 2.0 and FC = H were selected as the optimized parameter combination. The average results of ten replicates were presented in this study to ensure the reliability of the predicted models (Sillero & Barbosa, 2021). However, due to the fact that soil and UV‐B variables are absent in past and future periods, this study assumes that these variables are consistent across different time periods, which may affect the accuracy of the MaxEnt model. Meanwhile, the species distributions are affected by both biotic and abiotic factors, and only abiotic ones were considered in this study, which may also impact the accuracy of the model (Kuo et al., 2014; Xie et al., 2022). Therefore, in the future, the study should integrate more environmental layers and abiotic factors in order to obtain more accurate outcomes of the suitable distribution areas of species.

### 4.2 Contribution of environmental variables to species distribution models

Temperature and precipitation exert profound influences on the distribution of *A. costularis*. The results of percentage contribution rates showed that temperature (46.2%) contributed most to the *A. costularis* distribution, followed by precipitation (28.7%), soil factors (14.1%), and UV‐B (11.0%). According to the jackknife test, temperature annual range (Bio7) contributed most to the establishment of the distribution prediction model of the species. The MaxEnt model showed that temperature was the key factor determining the current distribution of *A. costularis*. *Alsophila costularis* is difficult to propagate in the wild because its spore germination is impacted by environmental factors, including temperature and moisture (Cheng et al., 1990; Pu et al., 2023). Ultraviolet radiation can cause injuries to aboveground organs of organisms and limit their distribution (Wu et al., 2021). Tree ferns generally grow within light gaps in the canopy to avoid the effects of Ultraviolet (Wei et al., 2021). Of the five most important environmental factors, temperature annual range was a decisive factor (contribution rate of 44.9%). Especially, we revealed the optimum growth conditions as follows: temperature annual range of 13.74–22.44°C, precipitation of warmest quarter greater than 668.33 mm, mean UV‐B of highest month of 5653.06–7338.84 J/m^2^/day, precipitation of wettest month greater than 277.71 mm, and precipitation of coldest quarter of 45.84–92.96 mm.

Currently, *A. costularis* is mainly distributed in Yunnan Province rather than in other regions. The unique subtropical climate in Yunnan provides suitable conditions for spore germination of *A. costularis* and promotes the spread of spores. Taiwan Island and Hainan Island were identified as suitable habitats for *A. costularis*, but we did not find occurrent records in these two islands, suggesting that these areas could be used as introduction area for *ex situ* conservation measures.

As a relict fern, *A. costularis* experienced drastic environmental changes, especially climatic fluctuations. Correspondingly, its suitable habitats also changed over time. In the LIG, the distribution of *A. costularis* was probably restricted to southern Yunnan and Hainan, which had a range of 25.69 × 10^4^ km^2^. In the LGM period, under the influence of drier and colder climatic conditions (Nogués‐Bravo et al., 2010), its suitable area contracted toward southwestern China, which was identified as a climatically stable area over time. In summary, the suitable habitat of *A. costularis* showed a trend of contraction toward southwestern Yunnan during the glacial period, with most of the remaining habitat lost. The diverse topography of southwestern China creates a great variety of habitats, which supports the continued survival of species. As a result, southwestern China has been identified as a climatically stable refugia (Tang et al., 2018). The multi‐plateau and mountains also could have provided refuges for *A. costularis* in Quaternary glaciers (Huang, 2004), which has been confirmed in other plants, such as *Sphaeropteris brunoniana* (Wang & Guan, 2011), *Bretschneidera sinensis* (Hu et al., 2017), *Saruma henryi* (Zhou et al., 2010), *Cathaya argyrophylla*, *C. spinulosa*, *Davidia involucrate*, *Eomecon chionantha* (Li, 1998), and *Pinus armandii* (Liu et al., 2014). Afterward, with the warming of temperature, *A. costularis* expanded to northern Yunnan, western Guangxi and southern Guangdong. Furthermore, the differences between the current and past distributions indicated that *A. costularis* likely experienced distribution range changes over short periods of time (Ramírez‐Barahona & Eguiarte, 2014). In the future, the mean surface temperature will increase by 1.1–6.4°C at the end of the century, which provides climatic conditions for the continued northward expansion of *A. costularis* (Gao et al., 2023). Therefore, compared with the current period, the suitable habitat of *A. costularis* mainly expanded toward northern Yunnan and southwestern Guizhou under four different SSPs, with an expansion area greater than 21.92 × 10^4^ km^2^. The expansion of the suitable area is greatest in the 2090s under the SSP1‐2.6 scenario, reaching 43.38 × 10^4^ km^2^. With future climate change, southwestern Yunnan remains a climatically stable area. The centroids of species generally remained unchanged over time.

The MaxEnt predictions showed that the core suitability areas of *A. costularis* are mainly focused in southwestern China, especially in Yunnan Province. Although there is currently no report on the spore of *A. costularis*, we infer that its spores lost viability quickly at room temperature as a member of Cyatheaceae (Li et al., 2010). Abundant rainfall in tropical and subtropical habitats guarantees their rapid germination and meets the demand of spermatozoids for water, especially in June when spores mature (Peck et al., 1990). Unique humid climate in Hainan Island facilitates the spread of fern spores by means of monsoon and rainy season with storm and typhoon (Wang et al., 2016).

The actual distribution of species is influenced by a variety of factors, such as biotic and abiotic factors. However, species distribution models take into account only a portion of the environmental variables, without considering historical and biotic factors, which may affect the accuracy of the predictions (Kuo et al., 2014; Xie et al., 2022). This study assumes that the soil and UV‐B variables are consistent between the historical and current periods due to the lack of historical data, which can lead to less environmental change in the historical period, which in turn affects the models' outcome. Biotic factors can affect the distribution of species, such as competition and predation (Gao et al., 2023). Accurate prediction of species distribution models requires more comprehensive and better distributed occurrence records of species (Feeley & Silman, 2011; Hernandez et al., 2006). Meanwhile, ecological interactions, dispersal capacity, and habitat preferences all affect the accuracy of modeling results (Haq et al., 2023).

### 4.3 Conservation and management of *A. costularis*

Climatic change exerts a huge impact on plants, which is a challenge for their conservation due to limited migration (Nunes et al., 2022; Varol et al., 2022). The survival and sustainable development of plants directly depend on the availability of suitable habitat (Shen et al., 2022). In this study, the distribution of *A. costularis* is predicted to extend toward northern Yunnan and western Guizhou in the future. More attention should be given to *A. costularis* in the context of global warming based on its fidelity to humid climates and habitats. We should strengthen *in situ* conservation in the current suitable range and establish more nature reserves and small protected area (Gao et al., 2022; Xiao et al., 2019). *Alsophila costularis* is vulnerable to soil pollution, so the uncontaminated ravine forests should be treated as the priority protected areas in Yunnan (Xiao et al., 2019). Based on growth condition of *A. costularis* population, protected sites could be delineated to sustain fern flourishing in the natural habitats. The *ex situ* conservation is another effective protection strategy for *A. costularis*. Due to the slow natural migration, species can be transported to the new added suitable habitat predicted by MaxEnt from the damaged habitat through assisted migration (Gómez‐Pineda et al., 2020; Hällfors et al., 2016). Assisted migration may disturb the ecological balance in the migratory site due to the potential of biological competition, and it is therefore recommended that specific areas be designated for migratory species (Bai et al., 2018). In addition, populations at introduced sites should be dynamically monitored and managed to ensure higher survival rates. The protected areas within the stable areas were found in Xishuangbanna Dai autonomous prefecture. Protection of areas other than this region in southwestern Yunnan, as well as Hainan and Taiwan Islands and Guangdong, should be strengthened to cope with future climate change. Our study provides a valuable information for the distribution and conservation of *A. costularis*.

## 5 CONCLUSION

An ecological niche model was used to explore the impact of climate change on the geographical distribution of *A. costularis* and found that current suitable areas were mainly concentrated in southwestern and southern China. It was predicted that in the future, *A. costularis* would significantly expand to northern Yunnan and western Guizhou, with increases in the suitable areas ranging from 21.92 × 10^4^ km^2^ to 43.38 × 10^4^ km^2^ under different SSPs. The stable areas were located in southwestern Yunnan.

Southwestern China, including Yunnan Province, presents the core suitability area for *A. costularis*. The key environmental factors determining the potential distribution of *A. costularis* are temperature annual range, precipitation of warmest quarter, mean UV‐B of highest month, precipitation of wettest month, and precipitation of coldest quarter. The environmental variables contribute to understand specific growth conditions of *A. costularis*, especially its preference for the warm and humid climate and improving the management and conservation. The accuracy of the MaxEnt model is affected by the choice of variables and species occurrence records, and more environmental factors and more comprehensive occurrence records should be integrated in future studies to obtain more precise predictions.

## AUTHOR CONTRIBUTIONS

**Zhen Wang:** Conceptualization (equal); methodology (equal); software (equal); writing – original draft (equal). **Ning Li:** Formal analysis (lead); methodology (equal); software (equal); writing – original draft (equal). **Ruixiang Xu:** Methodology (equal); software (equal). **Zhanming Ying:** Data curation (equal). **Xiaoxian Ruan:** Data curation (equal); software (equal). **Ting Wang:** Conceptualization (equal); funding acquisition (equal); project administration (equal); supervision (equal). **Wenbo Liao:** Conceptualization (equal); supervision (equal). **Yingjuan Su:** Conceptualization (equal); funding acquisition (equal); project administration (equal); writing – review and editing (equal).

## FUNDING INFORMATION

This work was supported by the National Natural Science Foundation of China (31872670 and 32071781), Guangdong Basic and Applied Basic Research Foundation (2021A1515010911), Science and Technology Projects in Guangzhou (202206010107), Project of Department of Science and Technology of Shenzhen City, Guangdong, China (JCYJ20190813172001780 and JCYJ20210324141000001), and Research Project of the Reform about Teaching Method and Skills from Sun Yat‐sen University and Guangdong Province.

## CONFLICT OF INTEREST STATEMENT

The authors declare no conflict of interest.

## Supporting information


Appendix S1.

## Data Availability

The data analyzed or presented in this study are openly available in Dryad (https://doi.org/10.5061/dryad.8gtht76xf).
