# Supplementary material for: Distribution model and prediction of the tree fern Alsophila costularis Baker (Cyatheaceae) in China
Source: Ecol Evol. 2024 Jun 21;14(6):e11594. doi: 10.1002/ece3.11594 (PMC11192646; doi:10.1002/ece3.11594)
Supplement: Supplementary file 1 — Appendix S1. [file ECE3-14-e11594-s001.docx]

Supplementary materials


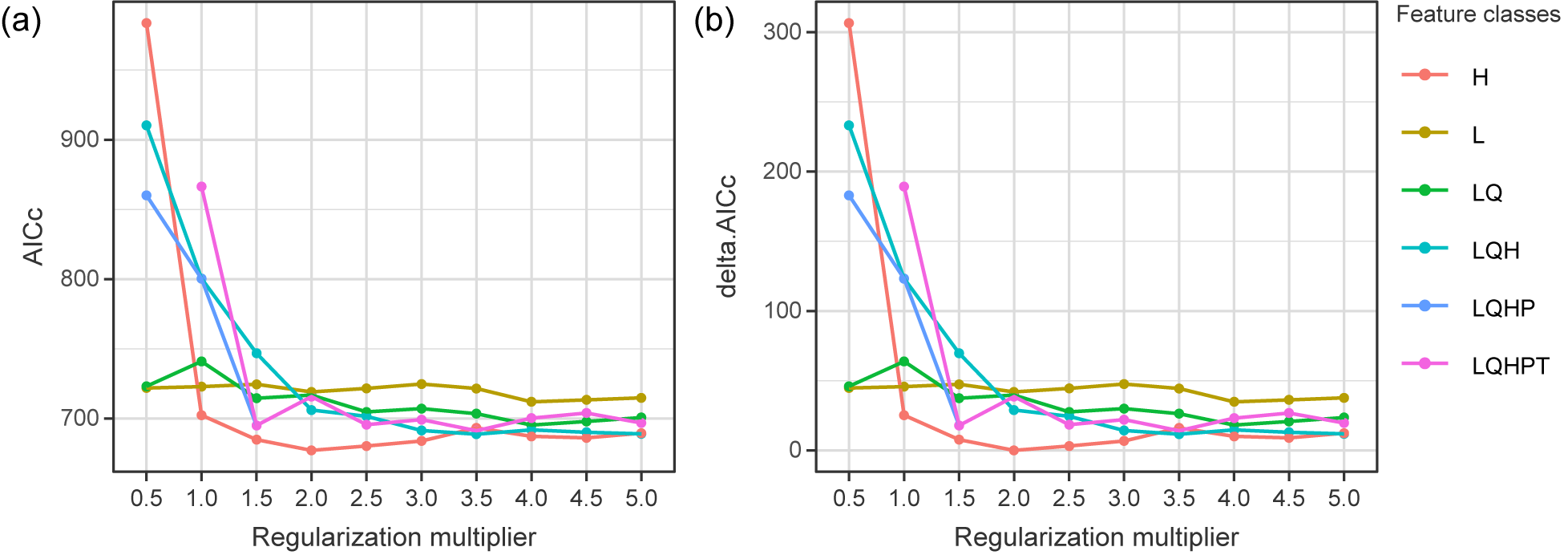


**FIGURE S1** The performance of MaxEnt model under different parameter combinations. (a) AICc and (b) delta.AICc. L = linear, Q = quadratic, H = hinge, P = product, and T = threshold.


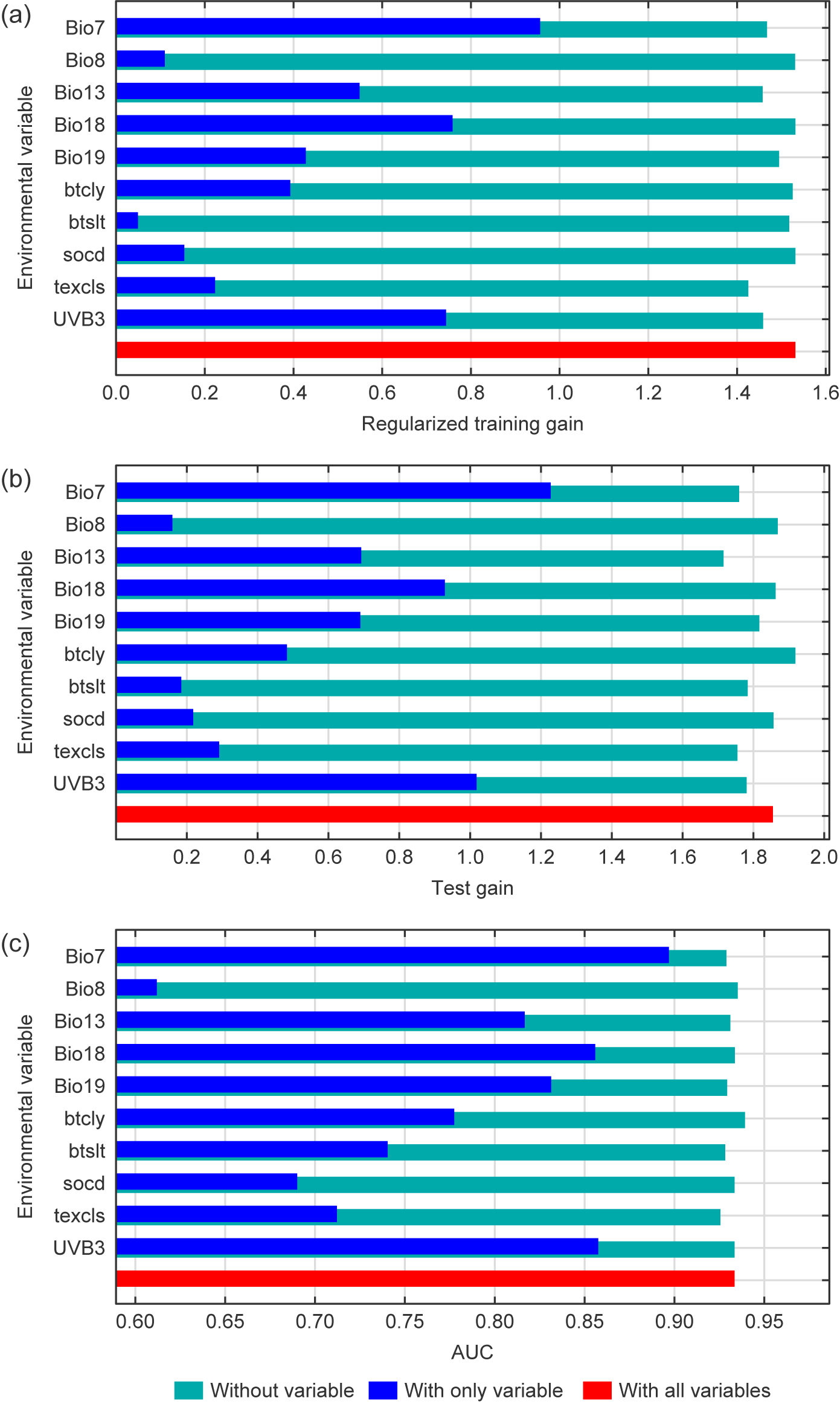


**FIGURE S2** Assessment of ten environmental variables contribution using the jackknife test of regularized training gain (a), test gain (b) and AUC (c). Blue bars demonstrate contribution of only one variable; green bars, without variable; red bar, with all variables.


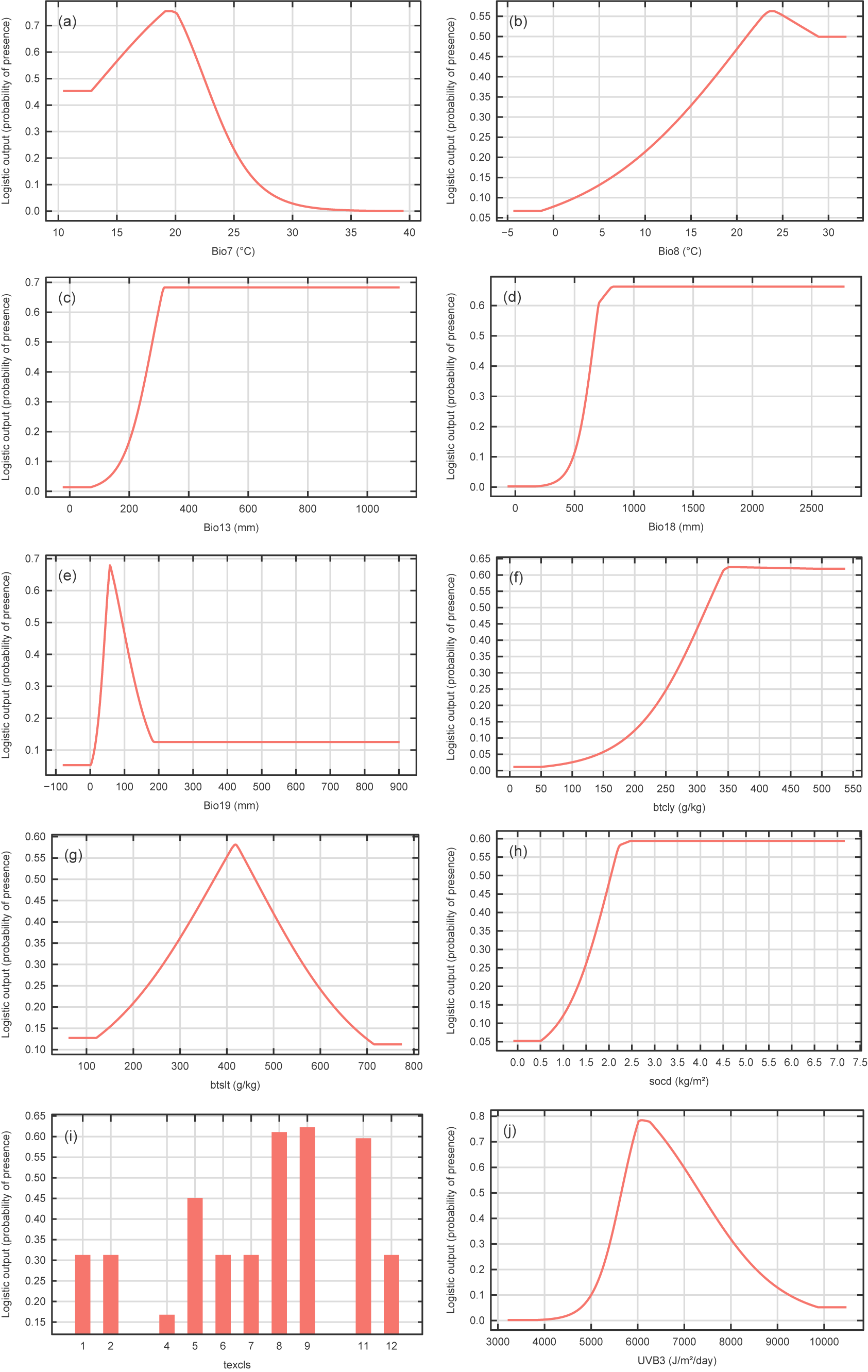


**FIGURE S3** Response curves of *Alsophila costularis* to the ten environmental variables. (a) Bio7, (b) Bio8, (c) Bio13, (d) Bio18, (e) Bio19, (f) btcly, (g) btslt, (h) socd, (i) texcls, and (j) UVB3. The edges of the red curves or histogram exhibit the mean response.
